# Social cognition and metacognition in great apes: a theory

**DOI:** 10.1007/s10071-022-01662-0

**Published:** 2022-08-01

**Authors:** Michael Tomasello

**Affiliations:** 1 Duke University, Durham, North Carolina USA; 2 Max Planck Institute for Evolutionary Anthropology, Leipzig, Germany

**Keywords:** Great apes, Metacognition, Social cognition, Theory of mind

## Abstract

Twenty-five years ago, at the founding of this journal, there existed only a few conflicting findings about great apes’ social-cognitive skills (theory of mind). In the 2 ½ decades since, we have discovered that great apes understand the goals, intentions, perceptions, and knowledge of others, and they use this knowledge to their advantage in competitive interactions. Twenty-five years ago there existed basically no studies on great apes’ metacognitive skills. In the 2 ½ decades since, we have discovered that great apes monitor their uncertainty and base their decisions on that, or else decide to gather more information to make better decisions. The current paper reviews the past 25 years of research on great ape social cognition and metacognition and proposes a theory about how the two are evolutionarily related.

In the 1960s Psychology experienced a cognitive revolution. The result was what came to be called Cognitive Science, a multidisciplinary field including psychology, philosophy, linguistics, neuroscience, and artificial intelligence. The study of cognitive processes in non-human animals did not play a significant role in this early history. This was, first, because much of the early work in cognitive science was focused on language and the concepts and knowledge that could be represented in language and, second, because the study of animal behavior at the time was dominated by Behaviorism and Ethology, neither of which was much interested in cognitive processes. Even today, most textbooks on cognitive science have more coverage of artificial intelligence and robotics than of animal cognition.

The breakthrough came in the late 1970s with the publication of two papers in the journal *Behavioral and Brain Sciences* that served to connect the study of animal behavior with human cognitive development. First, Parker and Gibson (1979) proposed a theory of the evolution of human cognition and language that used Piaget’s developmental theory as the overarching framework, supporting their account with experimental studies of various primate species on such things as object permanence, tool use, and spatial cognition. Second, Premack and Woodruff (1978) reported a study that asked the question” Does the chimpanzee have a theory of mind?”, which later helped to spawn a vast body of work on the development of human children's skills of social cognition. The connection to developmental psychology was crucial in incorporating animal behavior into the cognitive revolution because developmental work was mostly not language-centric. This developmental perspective was central to Tomasello and Call’s (1997) account of primate cognition, which they characterized as comprising skills for understanding the physical world (based on Piaget’s account of human children’s understanding of space and objects, tools and causality, and quantities) and skills for understanding the social world (based on work in cognitive-developmental psychology on human children’s cooperation, communication, social learning, and theory of mind).

The first issue of *Animal Cognition* was published 1998, and the authors of this 25th anniversary issue have been asked to choose an area of research and summarize the progress made in these last 2 ½ decades. I have chosen an area descending directly from Premack and Woodruff’s original (1978) paper: great ape social cognition (theory of mind). In the context of an historical account of progress in the field, I outline a theory of how great apes have come to have such remarkable social-cognitive skills. The key in my account is their connection with another set of skills, namely, skills of metacognition in which individuals executively monitor their own cognitive processes as they seek to make effective behavioral decisions. The claim will be that great ape skills of social cognition and metacognition are intimately interrelated both evolutionarily in species and ontogenetically in individuals.

## Status Quo 1997

Because our book *Primate Cognition* was published at around the same time as the first issue of *Animal Cognition*, it provides a well-documented starting point for the *status quo ante*. The question in 1997 was not whether great apes could predict the behavior of others based on various behavioral cues—that was clear—but whether in doing so they operated with an understanding of the underlying mental states involved. In addition to naturalistic observations (e.g., de Waal 1982; Byrne and Whiten 1988), by 1997 researchers had developed several ingenious experimental paradigms to investigate questions of great ape social cognition. Already at this early date, there were experimental studies of how great apes understand (i) goals and intentions, and (ii) perception and knowledge.

With regard to goals, in their original study Premack and Woodruff (1978) presented a single, language-trained chimpanzee with pictures of humans struggling with problems, for example, opening a shuttered door. She could choose among an array of other pictures potentially suggesting a solution, for example, a picture of a key. She did this reasonably well, but it was only a single, language-trained ape and she might have just been relying on an association between doors and keys. Then, Povinelli et al. (1998) gave chimpanzees the opportunity to distinguish intentional from accidental actions, and they basically failed to do so: they had no preference for a human who spilled their juice accidentally from one who poured it out onto the floor intentionally. And Tomasello (1996) reviewed a number of studies of great ape social learning and concluded that there was no good evidence that individuals understood the goals and intentions of the agents performing the actions; when learning from social models they were simply using their own strategies for reproducing environmental results (emulation).

With regard to perception, a number of studies provided evidence that chimpanzees understood when others were looking at or attending to them, for example, they used visually based gestures only when others were already looking at them (Tomasello et al. 1994). However, Povinelli and Eddy (1996a) found that chimpanzees did not seem to distinguish between more subtle differences between different recipients of a communicative gesture involving the eyes in particular, for example, they did not distinguish between the visual capabilities of a human who wore a blindfold over his eyes from one who wore one over his forehead. Povinelli and Eddy (1996b) found that chimpanzees followed a human’s gaze direction, but again they did not seem to understand the role of the eyes in the process.

With regard to knowledge, Povinelli et al. (1990) and Povinelli et al. (1994) reported a series of studies in which chimpanzees were sometimes successful and sometimes unsuccessful in attempting to distinguish between a human experimenter who was knowledgeable about the location of food from one who was ignorant. Again, they did not seem to understand the role of the eyes in particular in humans’ potentially coming to have knowledge about a situation. Woodruff and Premack (1979) conducted a study in which chimpanzees had the opportunity to mislead a human experimenter—enabling them to obtain some otherwise inaccessible food—and they learned to do so but only after many dozens of trials, suggesting that they may have just been learning to manipulate the behavior of the human communicatively in profitable ways. The same behavioral interpretation is also possible for the anecdotal observations of nonhuman primate “deception” reported by Menzel (1974) and Byrne and Whiten (1992).

On the basis of these experimental studies, as well as other observational studies, Tomasello and Call (1997, p. 329) concluded that “There is currently no solid evidence that nonhuman primates understand the intentionality or mental states of others”. To be clear, they claimed that many nonhuman primates understood much about the actions of others, for example, they understood who in their group was dominant to whom and who was friendly or not friendly with whom. And these same primates could predict the behavior of others based on a knowledge of how they had behaved in such situations in the past, the current cues present in the environment, and so forth. But they were seemingly not doing this based on an understanding of any underlying intentional or mental states. Interestingly and importantly, almost nothing was known in 1997 about the executive or metacognitive capacities of great apes or any other nonhuman primates.

In the 25 years since *Primate Cognition* was published significant progress has been made on all of these research topics. In what follows, I look first at great ape skills of social cognition and then at great ape skills of metacognition. I conclude with a proposal about how the two might be related evolutionarily.

## Great ape social cognition

Few areas of research in primate cognition have seen more progress in the last 25 years than the study of great ape social cognition (theory of mind). We look first at how great apes understand perception and knowledge and then at how they understand goals and intentions (reflecting the historical order of progress).

### Understanding perception and knowledge

Studies of great ape gaze following are suggestive of an understanding of the perception of others (see Call and Santos 2011), but they are not definitive because the individual’s response—looking in the same direction as another—could just be a response to a cue. More diagnostic are studies in which the individual must engage in an action that demonstrates that they understand the *content* of what the other perceives.

In a series of five studies, Hare et al. (2000) placed a subordinate and a dominant chimpanzee into rooms on opposite sides of a third room. Each had a guillotine door leading into this middle room which, when opened at the bottom, allowed them to observe two pieces of food at various locations—and to see the other individual looking under her door. After the food had been placed, the doors for both individuals were opened, and they were allowed to enter the middle room. The basic problem for the subordinate in this situation is that the dominant will take all of the food she can see. However, in some cases things were arranged so that the subordinate could see one piece of food that the dominant could not see, for example, by placing it on the subordinate’s side of a small barrier (with the other piece of food in the open). The question was, thus, whether the subordinate knew that the dominant could not see a particular piece of food, and so it was safe to go for it. The basic finding was that the subordinates did indeed go for the food that only they could see much more often than they went for the food that both they and the dominant could see. Importantly, there were no differences between the early trials and later trials, thus ruling out that individuals were learning during the experiment the most effective way to get the food. One possibility is that subordinates in these studies may have been monitoring the dominant’s behavior, rather than their perceptual access to the food, and reacting to that. But this possibility was ruled out by giving subordinates a small headstart and forcing them to make their choice between the two pieces of food before the dominant was released into the middle room. Moreover, in an additional control condition the dominant’s door was lowered before the two competitors were let into the room (and again the subordinate got a small headstart), so that the subordinate could not see the dominant at all at the moment of choice—and so could not react to her behavior—and subordinates still targeted the piece of food the dominant could not see. In still another control condition food was placed on the subordinate’s side of a transparent barrier, and subordinates—understanding that the dominant's view of the food was not blocked by this clear barrier—chose equally between the two pieces of food.

In addition to discerning what a competitor could and could not see, in another set of studies great apes attempted to actually manipulate what a competitor could and could not see—a design which eliminates associative learning of cues as an alternative explanation because the subject is actually implementing an active behavioral strategy. Hare et al. (2006) and Melis et al. (2006) had chimpanzees compete with a human (sitting in a booth) for two pieces of food. In some conditions, the human could see the ape equally well if it approached either piece of food (one on each side of the booth). In these cases, the ape had no preference for either piece. But in the key condition, a barrier was in place so that the apes could approach one piece of food without being seen. And this is exactly what they did. They even did this in a variation in which the choice confronting them was to reach for food from behind a barrier (such that the human could not see their body) but either through a clear tunnel (where the human could potentially see their reaching arm) or an opaque tunnel. They imagined what the human could see of their reaching arm. In a follow-up study, these same individuals preferentially chose to pursue food that they could approach silently—so that a distracted human competitor could not hear them—as opposed to food which involved making noise en route. This generalization to a completely different perceptual modality—audition versus vision—speaks to the power and flexibility of the cognitive skills involved.

A further question now was whether great apes are able to discern not only what another individual sees but also what he knows, in the sense that he has perceived something in the past in a way that still influences his behavior. Hare et al. (2001) thus used the conspecific food competition paradigm of Hare et al. (2000), but with only one piece of food. The food was always hidden from the dominant behind one of two barriers (which the subordinate subject could always see). The key manipulation was that the dominant either did or did not witness the hiding process (subordinates always saw the entire hiding procedure and could monitor the visual access of the dominant competitor as well). The main finding was that subordinates preferentially went for the food that dominants had not seen being hidden, whereas they stayed away from it if the dominant had witnessed the hiding process. They knew not only what the dominant could and could not see, but also what he had and had not just seen in the immediate past—and so knew. In another condition, just before the moment of choice the dominant individual was switched for another dominant individual (who had seen nothing); in this case subordinates now felt free to go for the food no matter what had transpired earlier, presumably based on their knowledge of what the particular individuals involved (the two dominants) had and had not seen previously—and so knew. This is important because it rules out the possibility that subordinates only used the mere presence/absence of a dominant, any dominant, during the baiting process as a behavioral cue.

In another experimental paradigm, Kaminski et al. (2008) ruled out the possibility that subordinate chimpanzees in the Hare et al. (2001) study were just avoiding the food that the dominant had seen at any time (the so-called “evil eye” hypothesis. They exposed chimpanzees to a back-and-forth game in which a subject and a competitor took turns choosing from a row of three opaque buckets, two of which contained food. The game began with a hiding event, which established one piece of food as a "known" to both subject and competitor—since both saw it being hidden in one of the buckets (and both saw the other watching)—and another piece of food as "unknown" to the competitor (but known to the subject)—since only the subject saw it being hidden in another bucket. The third bucket stayed empty. In the Competitor First condition, the competitor then got to make the first choice by selecting a bucket from behind an occluder, and then the subject got her turn to choose. If she had noted which of the two pieces of food the competitor had watched being hidden (the known piece), then in her choice she should avoid that one, since by the time of her choice it should be gone. In the control condition, the Subject First condition, the subject chose first. In this case, it was reasonable for the subject to choose either bucket that she knew to have food. The main finding was that in the Competitor First condition, when the competitor had already chosen first, subjects avoided the known food, presumably because they knew that the competitor knew its location and had already taken it (whereas in the control condition, subjects chose randomly between the buckets with food). The chimpanzees knew what their competitor knew (i.e., what he had seen in the recent past) and used this to predict what choice he would make. In still another impressive skill of social cognition, in a similar find-the-food game, chimpanzees knew that their competitor would choose a board that was lying slanted on a table (as if some food were underneath) rather than a flat board (under which there could be nothing); they knew what kind of inference he would make from the perceptual situation (Schmelz et al. 2011).

It is still controversial whether great apes understand beliefs as distinct mental states. Krupenye et al. (2016) report two studies suggesting that great apes understand others’ beliefs. But they used experimental paradigms that are controversial in developmental psychology because they do not require the organism to express its knowledge in effective actions. The studies focus only on individuals’ looking patterns, specifically, anticipatory looking. The understanding of a belief per se is an understanding that the individual’s current perspective on the situation is potentially different from the objective situation. But studies based on looking patterns do not involve in any way the organism referencing an objective situation, such that the individual’s perspective may be correct or incorrect. So-called explicit studies of false belief, requiring the individual to respond appropriately to another’s false belief, have consistently found negative results for great apes. Tomasello (2018) reviews this evidence and argues that an understanding of beliefs requires an individual to coordinate its perspective with that of another individual, as well as with the objective situation as best she can determine it. Great apes’ understanding of mental states does not involve this comparison but rather is essentially an understanding of what others are registering of the world perceptually.

A variety of different studies, using a variety of different experimental paradigms, thus suggest that great apes are able to understand in some situations what others see, hear, know, and infer. They use this understanding flexibly, especially in situations of competition, to predict what others will do. Whether or not they understand others’ beliefs is a controversial issue in need of further research.

### Understanding goals and intentions

Understanding what others see and know is only useful in social life if it enables the prediction of what others will do. But to predict what others will do in novel situations one needs, in addition, to know what their goal is.

Premack and Woodruff’s original (1978) study of one chimpanzee’s ability to discern the goals of others produced, as noted above, equivocal results. Call et al. (2004), therefore, took a different approach. A human experimenter gave a chimpanzee food repeatedly through a glass panel. Then, on some trials, he did not give it. The experimental manipulation was that sometimes he did not give it because he was unwilling whereas at other times he did not give it because he was unable. The methodological advance was that the failure to give the food was actually accomplished in several different ways within each of these two broad categories. Unwillingness was instantiated in three different ways: the experimenter either simply stared at the food on the table in front of him without giving it, he ate it himself, or he teased the ape with it. Yoked to each of these three unwilling actions were two unable actions that resembled its counterpart fairly closely behaviorally with respect to exactly how and where the food moved and exactly where the experimenter looked. Thus, yoked to the unwilling-teasing action were (i) an unable-clumsy action, in which the human dropped the food accidentally en route to the chimpanzee (and it rolled back to him) and (ii) an unable-trying action, in which he attempted unsuccessfully to force the food through a small hole in the glass (and then brought it back to himself). The point is that the unwilling and unable goals or attitudes were instantiated in ways that differed from one another on the surface rather starkly. And yet, beneath the surface, in terms of goals, the one thing in common to the three different unwilling actions was that the experimenter did not want to give the food to the ape, and the one thing in common to the six different unable actions was that the experimenter did want to give the food to the ape and was trying to do so. The main result was that the chimpanzees reacted similarly to the various unwilling actions by expressing in some way frustration or impatience, whereas they reacted similarly to the various unable actions by being patient. The obvious conclusion is that the apes’ differential reaction in the two experimental conditions was due to the chimpanzees understanding of the two different goals involved no matter how they were expressed behaviorally.

Other evidence that chimpanzees understand goals then came from studies aimed at other questions. For example, Tomasello and Carpenter (2005) found that three young, human-raised chimpanzees imitated not what a human actually did, but what he was trying but failing to do or what he did intentionally as opposed to accidentally. Also relevant are studies of chimpanzees’ helping behavior (e.g., Warneken et al. 2007; Yamamoto et al. 2012) showing that when chimpanzees see an agent trying and failing to reach a goal—for example, reaching for an out-of-reach object or trying to open a locked door—they often discern his goal and help him to achieve it. In another paradigm, Buttelmann et al. (2008a) found that all four great ape species knew which of two plastic eggs contained food when a human attempted unsuccessfully to open one of them (e.g., by banging it or biting at it) but simply manipulated the other one disinterestedly (and they showed no such preference when they knew ahead of time that the eggs were empty). Relatedly, Buttelmann et al. (2009) found that individuals from all four great ape species were able to predict a human’s actions based on his emotional expression toward different objects; that is, they predicted he would choose an object toward which he expressed positive emotions and not choose one toward which he expressed negative emotions.

In all of these studies, the behavior of the human experimenter changed in subtle ways when he was pursuing different goals. Buttelmann et al. (2012) conducted two studies in which a human’s actions during the test phase were completely identical in the experimental and control conditions; what differed was only the context leading up to those actions, which could, potentially, lead to two different interpretations of what the human was doing. In the first study, with all four great ape species, during the test phase the human—identically in the experimental and control conditions—twirled a piece of metal on top of a box that the ape knew contained food, an action that in some contexts could be seen as trying to open the box. The difference between conditions was that in the experimental condition the apes had previously observed the human manipulating locks and latches on tops of other boxes and then opening them (and giving the food from inside)—so setting up the expectation that he would be trying to open the final target box as well—whereas in the control condition they had previously observed the human simply manipulating locks and latches on tops of boxes without opening them (giving food then from his pocket). The main finding was that the apes tended to wait longer in the experimental than in the control condition—presumably because only in this condition did they see the human’s action as trying to open the box.

In the second study (with chimpanzees only), a human sat on a stool giving the subject food. Some meters away there was a second bucket with food from which the subject had previously received food as well. In the test phase of the experiment—identically in experimental and control conditions—the human stood up from his stool and turned his body in the direction of the second bucket. In this situation, chimpanzees quite naturally anticipated what the human was doing and, in the absence of other information, assumed that he was headed for the second bucket, and so they rushed there first in anticipation. The trick was that there was also an experimental condition in which something happened prior to the human standing up, for example, a call came from a walkie-talkie in the same direction as the second bucket, or another human threw a clipboard to the experimenter and it landed short (again, in the same direction as the second bucket). In this condition, subjects stayed longer in their current location, presumably because they understood that the human’s goal in standing up was not to go to the second bucket but rather to fetch the walkie-talkie or clipboard. They interpreted the exact same behavior differently depending on their understanding of the goal the human was beginning to pursue by standing up. We may thus say that whereas the unwilling-unable and intention-accident studies demonstrate an understanding of goals-in-action, these studies demonstrate an understanding of prior goals (i.e., goals operative prior to action).

Other evidence for the understanding of goals has come from studies examining great apes’ looking patterns when watching goal-directed actions. Myowa-Yamakoshi et al. (2012) found that when observing an agent’s reaching actions chimpanzees anticipate his external goal—by looking to the intended object in anticipation—in the same way as do human infants. Further in this direction, Kano and Call (2014) examined all four species of great apes’ looking patterns in a similar situation, but they controlled spatial variables. Thus, they found that apes proactively looked to the intended target of a human’s reaches independent of spatial location. That is, specifically, after learning that a human typically reached for a particular object, they anticipated him continuing to reach for that object even when that object changed locations (and another object was in the old location). In contrast, the apes did not make any predictions when viewing a mechanical claw performing the same action in the same situation.

Finally, there is a pair of studies suggesting that great apes may understand something about the action plans of others as they are pursuing goals, that is, their intentions. First, Buttelmann et al. (2007) found that human-raised chimpanzees imitated certain highly unusual actions on objects, like turning on a light switch with one’s foot. But when the human demonstrator seemingly was using his foot only because his hands were occupied, the chimpanzees (whose hands were not occupied) did not imitate this action. They seemingly understood why the human chose the unusual behavioral means that he did (he was constrained by his hands’ occupation) and that he would have chosen another behavioral means if his hands were free. The chimpanzees understood not only what the experimenter was trying to do, his goal, but also the rationality behind the choice of action plan toward the goal, which represents an understanding of his intention. Second, Buttelmann et al. (2008b) extended this methodology to all four great ape species (all mother-raised) in a tool choice paradigm. For example, they saw a human choose a tool either freely or when one of the tools was physically inaccessible, and then they had a choice of tool. Orangutans, but not the other apes, understood that when there were no constraints the agent was making his action plan for tool choice freely, based on the tool’s qualities, but when there were constraints the agent took account of these in formulating his action plan. Again, this tuning in to the rational decision making of others may be seen as representing an understanding of their intention.

Taken together, these various studies strongly suggest that great apes do not attend to just the behavior of others, but also to the goals and, perhaps to some degree, the intentions underlying and structuring that behavior. They use this understanding flexibly to predict what others will do.

### Summary

Predicting the behavior of a competitor flexibly in novel situations requires an understanding of them as agents who pursue their goals and attend to things in the environment that are relevant to their goal pursuit. The term ‘theory of mind’ is not really felicitous in describing these skills; if great apes have a theory (or an understanding), it is a theory of agentive action that includes various mental states as underlying causal factors. In any case, whatever we call it, it is fair to say that in the animal cognition community, after a period of controversy, there is now a general consensus that great apes understand others as agents whose actions are driven by their goals/intentions and perceptions/knowledge. This consensus is built not on any single study, but on a variety of studies using a variety of different methodologies which all come to the same basic conclusion.

## Great ape metacognition

Like other successful organisms, great apes are evolved to make good behavioral decisions, that is, decisions that are effective in goal pursuit. But, sometimes it is difficult to make a decision because of a lack of sufficient information about the alternatives, or because the alternatives are difficult to distinguish perceptually, in which case the organism feels some kind of uncertainty. Organisms that have some kind of executive access to this feeling of uncertainty can take it into account and attempt to make the best decision in the given situation. Various mammals and primates have shown the ability to monitor and deal with their uncertainty, but primates, and perhaps especially great apes, may have some special skills of so-called metacognition.

### Monitoring uncertainty: opting out

The classic opt-out task is a discrimination or memory task in which the individual has a choice: it may either solve a problem and get a large reward or, if it is anticipating failure, opt out and get a small reward for free. When the problem is easy, monkeys and a few other species typically choose to solve the problem and get the larger reward. But when the problem is difficult, and so failure is likely, they learn over many trials to opt out and go for the free smaller reward (e.g., Hampton 2001; Smith et al. 2010; see chapters in Beran et al. 2012, for different viewpoints).

The classic opt-out task typically takes many hundreds of trials for subjects to begin using the opt-out response strategically. Researchers have, therefore, chosen not to test great apes; in precise this experimental paradigm, but rather similar ones based on the same general logic. Suda and Call (2006) looked closely at the behavior of chimpanzees, bonobos, and orangutans in a cognitive task presenting varying levels of difficulty. In particular, they examined subjects’ tendencies to hesitate when the problem was of moderate difficulty. In general, when the problem was either extremely easy or extremely hard (i.e., the apes were either extremely successful or unsuccessful) subjects responded relatively quickly. But when the problem was of intermediate difficulty, they hesitated significantly. The investigators note that, unlike the opt-out task, this is a spontaneous measure that is not connected to rewards, and therefore, explanations in terms of associative learning and reward contingencies are not applicable. Allritz et al. (2021) strengthened this finding by observing that when chimpanzees were presented with difficult (versus easy) problems on a touchscreen, their hand more often hovered over the screen as a natural and endogenous expression of their uncertainty. From the other direction, Beran et al. (2015) gave chimpanzees a discrimination task for which they would be rewarded for correct performance, but from a reward dispenser that was some meters away with the reward only briefly available. When the discrimination was easy, they went quickly to the dispenser so as to get the reward while it was still available, whereas if the discrimination was difficult, they typically went more slowly or not at all. Their moving to the reward dispenser was governed by their confidence that they had chosen the correct answer.

Suda-King (2008) devised a more natural opt-out task for orangutans requiring little or no training. A large reward was hidden in one of two blue cups, and a small reward was placed (always in full view of the subject) in a yellow cup. If subjects saw the large reward being hidden, they chose the blue cup containing it. But if they did not witness the hiding process, they often opted for the safe choice, the yellow cup. Suda-King et al. (2013) replicated the same basic findings with gorillas, and Neldner et al. (2015) replicated them with both chimpanzees and young human children. Haun et al. (2011) presented this same basic task to all four great ape species but varied both the probability of success and the relative value of the risky versus the safe choice. They also attempted to manipulate subjects’ level of uncertainty by varying the amount of information available to them. They replicated the basic results of the previous studies—subjects went for the safe choice when they had not seen the hiding process—but, in addition, they found that subjects were more prone to choose the safe option when its value was increased and/or when the probability of success in the risky option decreased. Consistent with their foraging proclivities and previous research, chimpanzees and orangutans were more risk-prone, whereas bonobos were more risk-averse (with gorillas in between). But all species were seemingly monitoring something associated with the decision-making process—perhaps feelings of uncertainty for certain behavioral choices—to maximize their rewards in the situation.

### Monitoring uncertainty: seeking information

Experiments investigating the opt-out response to uncertainty suggest that many nonhuman primates know when their decision-making process is uncertain. But another experimental paradigm has gone beyond this simple response to see whether and how great apes actually diagnose the source of their uncertainty—typically, a lack of information—and seek to do something about it, for example, gather further information. Interestingly, research with several species of nonprimate mammal suggests that they do not engage in this particular type of executive or metacognitive monitoring (Roberts et al., 2012).

Call and Carpenter (2001) presented chimpanzees and orangutans with a situation in which they either did or did not see the process of food being hidden inside of one of the two opaque tubes. When they witnessed the hiding process, subjects of all three species chose a tube more or less immediately. But when they did not witness the hiding process, they went to some trouble to bend their bodies down to look into the tubes to discover where the food was located before choosing. The apes knew when they did not know, or at least when they were uncertain, but in this case they reflected on the decision-making process to identify the problem and fashion a solution. However, it is also possible to interpret these results as a function of a simple search strategy not involving metacognition: when I see the food’s location, I go for it, and when I do not see the food’s location, I search for it. But subsequent studies have rendered this simple alternative hypothesis insufficient.

Call (2010) followed up on these results with all four great ape species. He replicated the basic result with all species but went beyond it in several important ways. First, he found that the apes were more likely to seek extra information if the reward was highly valuable or if it had been longer since they acquired information about its location—both variables that also affect human decision-making in search tasks. Moreover, he found that if apes heard food as it was being placed inside of one of the tubes (E shook it), they did not seek further information because they already had the needed information from another source. Also, if they looked in the empty tube and saw no food, they immediately chose the other without looking (reasoning by exclusion), again because they had the needed information already. Apes’ behavior in both of these task variations mirror human behavior in the same situations (see Call 2010, for references). In a further variation on this theme, Bohn et al. (2017; see also Mulcahy 2016) found that when apes needed a tool of a certain type and did not know its location, they would seek that information before acting. In a series of studies with orangutans, Marsh and MacDonald (2012a, 2012b) again found that the apes looked for information only when it was needed, dispensing with looking into the tubes when they could infer its location (by exclusion). Moreover, they searched for information most often when it was energetically cheapest, when the odds of making an error were greatest, when the reward was largest, and when the benefit/risk trade-off was most favorable. Their information seeking was thus highly flexible, tailored to the particularities of the situation. In general, across all of these studies, great apes were extremely flexible and skillful at diagnosing what information they were missing and how best to obtain it.

Finally, O’Madagain et al. (2022) gave chimpanzees, bonobos, and orangutans the opportunity to visually locate the best food in a situation at location X. The apes did this, indicating their belief by choosing that location (though not receiving the food as a result). Then, they were exposed to new information that called their initial belief into question: the new information suggested that the best food might be in location Y. The apes had the possibility at this point to seek further information (or not) that could either confirm or disconfirm their initial belief. Many apes then actively sought more information to resolve the discrepancy between their original belief and the new information by looking again into location X to check their initial judgment a second time. The apes were self-monitoring and controlling their decision-making after they had already made an initial decision to make the best final decision; they were reflecting on their initial decision in the light of newly obtained information and discerning the need to possibly revise that decision. For many philosophers, examining one's own knowledge critically and adjusting it in the light of new evidence, is best called rational decision making.

### Summary

Great apes are, thus, capable not just of monitoring and controlling their behavior but also of monitoring and controlling their decision-making metacognitively (see Carruthers and Williams 2022, for the view that they are not monitoring cognitive states per se, but rather certain feelings such as uncertainty and effort). Tomasello (2022) proposed that we should think of such metacognitive monitoring as involving a second tier of executive processes, what he calls reflective processes, requiring metacognition. Many mammalian species sometimes feel uncertain about a decision, and their subsequent decision making takes that uncertainty into account. But great apes can go beyond simple uncertainty monitoring to determine, at least in some cases, that the cause of their uncertainty is a lack of some relevant piece of information—they monitor and troubleshoot the decision-making process—and devise an appropriate strategy to ameliorate the problem. And, finally, they can even monitor their own beliefs about a situation metacognitively and make adjustments in the light of new information.

## A theory

Advocates of a so-called meta-representational approach to social cognition (the locus classicus is Perner 1991) have long pointed out that understanding one's own mental states and understanding the mental state of others are potentially the same or similar processes. Simulation accounts of mental state understanding are explicit in this regard, positing that human individuals use their own mental states to understand others’ mental states by explicitly simulating them.

Tomasello (2022) uses this insight to propose an evolutionary account of the origins of great apes’ social-cognitive and metacognitive skills. The basic idea is this. Executive monitoring of one’s own behavior is common among mammals. This is accomplished with a single tier of executive functioning that supervises the operational level of perception and action, and does such thing as inhibit prepotent responses when they are not appropriate. Great apes, in addition, engage in executive monitoring of their own mental decision making involving various mental processes such as goals and perceptions, as just reviewed. This is accomplished with a second-order tier of executive functioning—metacognitive or reflective functioning—that supervises the first-order executive tier. In the context of social competition in which understanding the mental states of others would be helpful, at some point in their evolution great apes, if not another primates, attributed the same mental states that they were monitoring in themselves to others. The reason that primates, and perhaps especially great apes, have evolved to make such attributions is because of increased food competition relative to other mammals. In particular, a focus on fruit, as a patchily distributed clumped resource, leads to strategies by subordinates to get around dominants monopolizing the fruiting tree (see Tomasello 2022, for a review).

It is possible that important in this evolutionary process as well were skills of social learning or imitation in which individuals were required to establish a correspondence between the behavior of others and their own behavior. Great apes’ special skills of social learning in comparison with those of other mammals and primates might suggest why they are particularly skillful at understanding the mental states of others. Once again, the relevant ecological conditions may be increased food competition. In this case, individuals would be able to obtain more and better food—including in the context of habitual tool use, which is mostly confined to great apes among primates—if they could exploit the behavioral strategies of others for their own benefit (again see Tomasello 2022, for a review).

One study in particular provides important evidence for apes’ ability to use their own mental states to understand those of others. Karg et al. (2015) had chimpanzees compete with a human for food. Experimenters arranged things so that a chimpanzee subject first experienced a situation in which it could see through a screen lid on a box to detect what was inside. Another box had an opaque lid. The boxes were then relocated and re-oriented, so that now from the ape’s new slanted viewing angle, the screen lid (as well as the other lid) was opaque—but there were holes underneath so that she could actually see inside both boxes. The human competitor then approached the boxes and looked straight into them, from the viewing angle that the chimpanzee subject had used originally. When the two of them now competed for the food inside the boxes, the chimpanzees knew that the human could see through the screen lid to the food inside even though she herself could not at that moment—so she should not go for that food but rather the food in the other box. The only way the subject could know this was from her own previous experience of having looked directly through the screen lid into the box from the original viewing angle, which she was now attributing to the human. Importantly, Kano et al. (2019) report similar findings using other ape subjects and an anticipatory looking version of this same basic method (see also Schmelz et al. 2013).

In all, then, current research on great ape social cognition and metacognition suggests that they have evolved somewhat special skills in both of these domains. An interesting further hypothesis is that they are related in the sense that their skills of social cognition derive from a confluence of their skills of metacognition and social learning, enabling an attribution of mental states to others. Ecological conditions that might have led to this different way of functioning are unknown, of course, but it could be that the especially intense food competition among early great apes led to greater skills of metacognition, social learning, and, ultimately, to their ability to use these skills to attribute underlying mental states to others and thereby to predict their behavior better in the context of intense food competition. This hypothesis would predict that across species (and possibly in ontogeny within a species) skills of social cognition and metacognition should be intimately related—and perhaps related to skills of social learning and to the intensity of food competition.

## Conclusion

The past 25 years have seen immense progress in the study of animal cognition, and *Animal Cognition* has been an important contributor to that progress. Because of Premack and Woodruff’s (1978) seminal explorations, the study of great ape social cognition has been one important strand of research in this overall program. We have gone from a situation in which we did not have experimental demonstrations of great ape social cognitive skills to a situation in which we have multiple experimental paradigms in which we can study various dimensions of these important skills. And the study of metacognition in great apes and other primates has added a critically important dimension to our understanding of how our nearest primate relatives understand various mental states and how they work. The hypothesis that great ape skills of metacognition and social cognition are intimately related is one that should be further explored in great apes and other species. More specifically, the current hypothesis would make the following five predictions, all of which could potentially be tested.

1. Across primate species—and perhaps across nonprimate species as well—there should be a correlation between self-regulatory and social cognitive skills.
2. Specifically, species that are skillful at behavioral inhibition should also be skillful at predicting and controlling the behavior of others.
3. Also, species that are skilful at metacognition (e.g., information seeking in the face of uncertainty) should also be skillful at the attribution of mental states to others, although this may be confined to species that are also skillful at social learning and/or behavioral imitation.
4. Species that have these correlated skills of self-regulation (or metacognition) and social cognition—at whatever level—should engage in more and/or more intense food competition than related species.
5. Possibly, though this is more speculative, the two sets of skills are interdependent during cognitive processing such that disrupting metacognitive monitoring—e.g., overburdening it with a distract or task—should negatively affect social cognitive skills in the moment.
